# Using anatomical landmarks to calculate the normal joint line position in Chinese people: an observational study

**DOI:** 10.1186/s13018-018-0963-2

**Published:** 2018-10-19

**Authors:** Aoyuan Fan, Tianyang Xu, Xifan Li, Lei Li, Lin Fan, Dong Yang, Guodong Li

**Affiliations:** 1 Department of Orthopedics, Shanghai Tenth People’s Hospital of Tongji University, 301 Yanchang Rd, Shanghai, 200072 China; 2 Tongji University School of Medicine, Shanghai, China; 3 Department of Radiology, Shanghai Tenth People’s Hospital of Tongji University, Shanghai, China; 4 Department of Neurosurgery, Shanghai Tenth People’s Hospital of Tongji University, Shanghai, China

**Keywords:** Knee joint line position, Total knee arthroplasty, Landmark, Computed tomography, Chinese population

## Abstract

**Background:**

Restoring the normal joint line (JL) is an important goal to achieve in total knee arthroplasty (TKA). We intended to study the veracity of several landmarks used to level the normal JL in Chinese people.

**Methods:**

Two hundred fifteen standard CT scans of knee joint were included to measure the distances from landmarks to distal JL (DJL) and posterior JL (PJL), along with femoral width (FW) in order to calculate the ratios. Landmarks included adductor tubercle (AT), medial epicondyle (ME), lateral epicondyle (LE), tibial tubercle (TT), fibular head (FH) and the inferior pole of the patella (IPP). Ratios were calculated between distances and FW (e.g. FHDJL/FW). Linear regression analysis and *t* test were used to determine the accuracy and the differences amongst sides of the leg, genders and races.

**Results:**

The average of IPPDJL/FW, TTDJL/FW, FHDJL/FW, LEDJL/FW, LEPJL/FW, MEDJL/FW, MEPJL/FW, ATDJL/FW and ATPJL/FW were 0.165, 0.295, 0.232, 0.297, 0.281, 0.327, 0.3PJL, 0.558 and 0.313, respectively. No significant difference had been found between the left and right leg. A gender difference was only found statistically on the ratio of IPP, and also, no linear correlation was observed only between IPP and FW. Most of the difference values lain in a 4-mm threshold for MEDJL (95.81%), LEDJL (94.88%), MEPJL (97.21%), LEPJL (94.88%), ATPJL (93.49%) and ATDJL (100%). Significant differences were observed amongst different races.

**Conclusions:**

AT, ME and LE can be used as reliable landmarks to locate the normal JL in Chinese population intraoperatively. It is meaningful to come up with a set of ratios to different races.

## Background

Restoring the normal joint line (JL) is an important yet challenging goal for surgeons to achieve in total knee arthroplasty (TKA) [1]. Malposition of the JL in the coronal plane happened frequently to primary and especially revision TKAs [2]. That malposition could cause relatively patella alta or patella baja [3], which may lead to unpleasant clinic outcomes. Researches showed that coronal changes of JL position may alter the patellar strain and the patellofemoral contact forces [3, 4]. Even 4–8 mm elevation or descent to the normal JL position could generate midrange flexion laxity, decrease to the patellofemoral contact area, which may lead to a lower total range of movement, postoperative knee pain, premature component wear and lower Knee Society Score [1, 5–8]. These outcomes may result in another revision TKA.

Anatomical landmarks such as adductor tubercle, medial epicondyle and lateral epicondyle were studied previously by many investigators. The absolute distances were measured from landmarks to JL, yet the distances may bias by different genders, heights or races [9]. In order to avoid those deviations, researchers began to use the ratios of absolute distances and femoral width [10]. Studies showed no statistical difference in ratios of different genders and heights [10, 11], yet we still need diverse ratios for various races. Literatures had proved that the Chinese population showed a modicum of anatomical difference [12–14], yet no study fully analyzes the usage of landmarks in the Chinese population.

With the benefits of ratios, studies showed a slight difference between different races [9, 15]. And several studies have already proved the distinction between the Chinese population and other countries [12–14]. Nevertheless, to our knowledge, no accuracy comparison amongst those osteal anatomical landmarks in Chinese population using computed tomography (CT) scan was published before. The purposes of our study were to (1) verify the non-gender otherness of ratios to Chinese people and compare whether left or right knee biased the ratios, (2) compare the accuracy of these landmarks in Chinese population, (3) provide ratios for surgeons to calculate the distance from several landmarks to JL and verify these veracity and (4) look out whether there is a difference amongst Chinese and other different races.

## Methods

A total of 215 standard CT scans(GE Medical Systems/LightSpeed VCT, Siemens, New York, USA) of knee joint from 194 patients(102 male and 92 female) examined in our hospital from January 2013 to July 2017 were collected in this study. No CT was performed solely for our study and no information which could be used to identify the patient was collected in our study. Our study was approved by the institutional review board of Shanghai Tenth Hospital Affiliated to Tongji University. CT scans that showed evidence of knee fracture, history of knee surgery, degeneration or malformation was excluded. Imaging data was derived from the PACS system along with patients’ age and gender. Patients younger than 18 or older than 40 years old were excluded from our study (average 30.56). Distances were measured by mimics 17.0 and analyzed by SPSS 20.0.

**Fig. 1:**
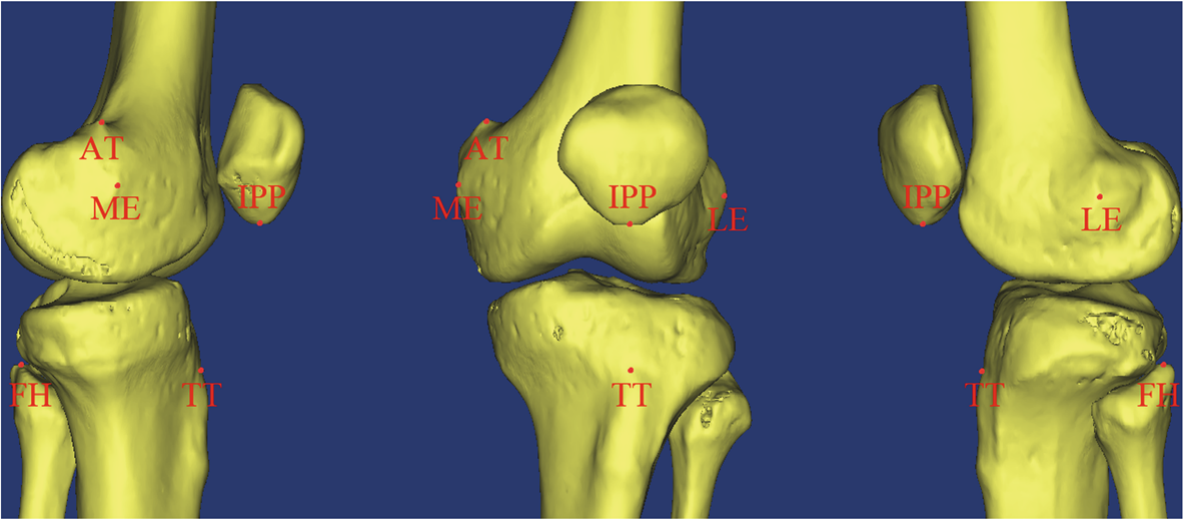
Positions of the knee joint landmarks. Landmarks were marked and verified on three-dimensional reconstruction. AT adductor tubercle, ME medial epicondyle, LE lateral epicondyle, TT tibial tubercle, FH fibular head, IPP the inferior pole of the patella

JL was defined as the tangent that connects two most distal points of the femoral condyles, but we used a plane to represent the JL so that measurement could take place on three-dimensional reconstruction. During measurements, we created a plane (distal JL, DJL) that crossed the JL and was vertical to the coronal plane as a reference to JL at full extension of the leg. We then created a plane (posterior JL, PJL) that is vertical to DJL and cross the two most posterior points of the femoral condyles as the reference to femoral JL under PJL degrees flexion of the knee for femoral landmarks. Selected anatomical landmarks were adductor tubercle (AT), medial epicondyle (ME), lateral epicondyle (LE), tibial tubercle (TT), fibular head (FH) and the inferior pole of the patella (IPP) (Fig. 1). In order to gather more accurate data, we came up with a bunch of methods based on previous researches [9, 16] and our pre-measurement. AT was measured at its most anterior and medial point where adductor muscle contacted with bone, which can be identified with the coronal plane reconstruction of the CT data. ME and LE were identified as the most prominent point of medial and lateral epicondyle, which can be located on transverse sections of CT. And landmarks were verified on computer-aided design (CAD) model created by three-dimensional reconstruction again (Fig. 1). TT is a rather large landmark, so after the pre-measurement, we decided to use the most proximal point of the slope of TT (Fig. 1). FH and IPP were measured on the most superior and inferior point and were easy to identify with the CAD model on three-dimensional reconstruction (Fig. 1). The perpendicular lines made from those landmarks to DJL were measured as the distance (mm), marked as ATDJL, MEDJL, LEDJL, TTDJL, FHDJL and IPPDJL. And we only measured the perpendicular distances from femoral landmarks to PJL, marked as ATPJL, MEPJL and LEPJL. The distance between the most prominent points of medial and lateral epicondyles was defined as the femoral width (FW). The ratios of distances between the landmarks to JL and FW were calculated respectively. We randomly chose 40 CT scans to measured the distances twice (day 1 and 2 weeks later) by two observers so as to determine intraobserver and interobserver variability before the measurements of all patients took place.

### Statistical analysis

Firstly, we used the data of those who underwent bilateral CT scan to determine whether there’s a difference between the two sides of the leg by paired *t* test. And then amongst all data, the unpaired *t* test was used to determine the difference between different genders and sides of leg again. Linear regression analysis was performed between FW and those distances separately. The average ratios were used to estimate the distance (e.g. ATDJL), and the difference value between the measured distance and estimate distance was calculated to verify the veracity of those ratios. Moreover, our data were compared with other investigators to determine the difference between Chinese and other populations using Student’s *t* test. A *p* value < 0.05 was considered statistically significant in this study.

## Results

### Intraobserver and interobserver variability

The measurements amongst intraobserver and interobserver did not statistically differ, demonstrating the methods’ reproducibility (Tables 1 and 2).

**Table 1:** Intraobserver measurements

	Measurements at day 1 (*n* = 40) (mm)	Measurements at day 14 (*n* = 40) (mm)	*p*
FW	82.39 ± 6.37	82.43 ± 6.33	0.668
IPPDJL	12.68 ± 6.55	12.88 ± 6.76	0.420
TTDJL	25.16 ± 2.68	25.06 ± 2.64	0.086
FHDJL	18.54 ± 4.25	18.85 ± 3.94	0.134
LEDJL	24.59 ± 2.87	24.70 ± 2.79	0.504
LEPJL	23.28 ± 2.29	23.44 ± 2.20	0.256
MEDJL	27.32 ± 2.71	27.48 ± 2.49	0.444
MEPJL	32.11 ± 2.63	32.15 ± 2.41	0.780
ATDJL	45.40 ± 3.36	44.86 ± 4.21	0.328
ATPJL	26.32 ± 2.18	26.20 ± 2.2	0.428

*FW* femoral width, *AT* adductor tubercle, *ME* medial epicondyle, *LE* lateral epicondyle, *TT* tibial tubercle, *FH* fibular head, *IPP* the inferior pole of the patella, *DJL* distance from landmarks to the distal joint line, *PJL* distance from landmarks to posterior joint line

**Table 2:** Interobserver measurements

	First measurer (*n* = 40) (mm)	Second measurer (*n* = 40) (mm)	*p*
FW	82.79 ± 6.26	82.63 ± 6.44	0.883
IPPDJL	11.59 ± 6.83	13.58 ± 6.31	0.245
TTDJL	25.23 ± 2.56	25.19 ± 2.76	0.739
FHDJL	18.07 ± 4.18	18.71 ± 3.99	0.432
LEDJL	24.96 ± 2.42	25.13 ± 3.18	0.787
LEPJL	23.28 ± 2.24	23.03 ± 2.24	0.497
MEDJL	27.82 ± 2.92	27.17 ± 2.19	0.253
MEPJL	32.31 ± 2.56	32.15 ± 2.49	0.702
ATDJL	45.59 ± 3.25	45.68 ± 4.30	0.902
ATPJL	26.41 ± 2.32	26.71 ± 2.09	0.396

*FW* femoral width, *AT* adductor tubercle, *ME* medial epicondyle, *LE* lateral epicondyle, *TT* tibial tubercle, *FH* fibular head, *IPP* the inferior pole of the patella, *DJL* distance from landmarks to the distal joint line, *PJL* distance from landmarks to posterior joint line

### Measured distances

Mean measurements of FW and the distances from landmarks to JL were given in Table 3. Measurements from different genders or sides of the leg were listed in Tables 3, 4 and 5 respectively. Data of both 21 patients who underwent bilateral knee CT scan and all 215 CT scans (116 left and 99 right) showed that there is no significant difference between the left and right legs on all the measurements (Tables 4 and 5). Compared with all the distances, the statistical difference had been found amongst different genders for except IPPDJL (*p* = 0.916).

**Table 3:** Mean measurements and gender difference

	Total (*n* = 215) (mm)	Male (*n* = 110) (mm)	Female (*n* = 105) (mm)	*p*
FW	79.61 ± 6.60	85.05 ± 3.84	73.92 ± 3.19	< 0.001
*IPPDJL*	*13.04 ± 5.16*	*13.01 ± 5.90*	*13.08 ± 4.26*	*0.916*
TTDJL	23.45 ± 3.74	25.32 ± 3.90	21.50 ± 2.31	< 0.001
FHDJL	18.48 ± 3.89	19.59 ± 4.34	17.32 ± 2.95	< 0.001
LEDJL	23.62 ± 2.70	25.12 ± 2.43	22.05 ± 1.97	< 0.001
LEPJL	22.37 ± 2.58	23.80 ± 2.31	20.86 ± 1.92	< 0.001
MEDJL	26.04 ± 2.83	27.65 ± 2.42	24.34 ± 2.18	< 0.001
MEPJL	31.05 ± 2.99	33.08 ± 2.26	28.93 ± 2.03	< 0.001
ATDJL	44.40 ± 3.76	47.39 ± 2.41	41.27 ± 1.91	< 0.001
ATPJL	24.89 ± 2.89	26.39 ± 2.64	23.31 ± 2.23	< 0.001

*p* was compared between genders

*FW* femoral width, *AT* adductor tubercle, *ME* medial epicondyle, *LE* lateral epicondyle, *TT* tibial tubercle, *FH* fibular head, *IPP* the inferior pole of the patella, *DJL* distance from landmarks to the distal joint line, *PJL* distance from landmarks to posterior joint line

**Table 4:** Mean measurements of 21 bilateral CT and sides of leg difference

	Total (*n* = 42) (mm)	Left (*n* = 21) (mm)	Right (*n* = 21) (mm)	*p*
FW	78.16 ± 5.71	78.07 ± 5.68	78.24 ± 5.88	0.338
IPPDJL	12.19 ± 4.59	12.36 ± 4.96	12.02 ± 4.31	0.602
TTDJL	21.12 ± 1.85	21.10 ± 1.90	21.15 ± 1.84	0.889
FHDJL	16.74 ± 3.80	16.84 ± 3.66	16.63 ± 4.03	0.526
LEDJL	22.74 ± 2.39	22.45 ± 2.50	23.04 ± 2.30	0.268
LEPJL	21.18 ± 2.41	21.10 ± 2.48	21.26 ± 2.39	0.640
MEDJL	25.40 ± 2.95	25.19 ± 3.28	25.61 ± 2.64	0.506
MEPJL	30.28 ± 2.42	30.19 ± 2.55	30.38 ± 2.34	0.421
ATDJL	43.18 ± 3.26	43.19 ± 3.38	43.16 ± 3.23	0.880
ATPJL	24.47 ± 2.54	24.57 ± 2.48	24.37 ± 2.66	0.367

*p* was compared between sides of the leg

*FW* femoral width, *AT* adductor tubercle, *ME* medial epicondyle, *LE* lateral epicondyle, *TT* tibial tubercle, *FH* fibular head, *IPP* the inferior pole of he patella, *DJL* distance from landmarks to the distal joint line, *PJL* distance from landmarks to posterior joint line

**Table 5:** Mean measurements of all CT and sides of leg difference

	Total (*n* = 215) (mm)	Left (*n* = 116) (mm)	Right (*n* = 99) (mm)	*p*
FW	79.61 ± 6.60	79.72 ± 6.99	79.49 ± 6.15	0.793
IPPDJL	13.04 ± 5.16	13.18 ± 5.18	12.88 ± 5.15	0.676
TTDJL	23.45 ± 3.74	23.52 ± 3.50	23.38 ± 4.02	0.787
FHDJL	18.48 ± 3.89	18.58 ± 3.82	18.36 ± 3.98	0.685
LEDJL	23.62 ± 2.70	23.82 ± 2.86	23.38 ± 2.49	0.223
LEPJL	22.37 ± 2.58	22.53 ± 2.79	22.18 ± 2.31	0.309
MEDJL	26.04 ± 2.83	26.01 ± 3.09	26.07 ± 2.51	0.862
MEPJL	31.05 ± 2.99	31.04 ± 3.19	31.07 ± 2.74	0.940
ATDJL	44.40 ± 3.76	44.72 ± 3.91	44.03 ± 3.57	0.177
ATPJL	24.89 ± 2.89	25.01 ± 2.92	24.75 ± 2.87	0.514

*p* was compared between sides of the leg

*FW* femoral width, *AT* adductor tubercle, *ME* medial epicondyle, *LE* lateral epicondyle, *TT* tibial tubercle, *FH* fibular head, *IPP* the inferior pole of the patella, *DJL* distance from landmarks to the distal joint line, *PJL* distance from landmarks to posterior joint line

### Ratios

The average ratios of all data and different genders were given in Table 6. No gender-specific difference had been found apart from IPPDJL/FW (*p* = 0.008).

**Table 6:** Mean ratios and gender difference

	Total (*n* = 215)	Male (*n* = 110)	Female (*n* = 105)	*p*
*IPPDJL/FW*	*0.165 ± 0.066*	*0.154 ± 0.071*	*0.177 ± 0.058*	*0.008*
TTDJL/FW	0.295 ± 0.040	0.298 ± 0.047	0.291 ± 0.031	0.199
FHDJL/FW	0.232 ± 0.046	0.231 ± 0.051	0.234 ± 0.040	0.526
LEDJL/FW	0.297 ± 0.024	0.295 ± 0.024	0.298 ± 0.024	0.371
LEPJL/FW	0.281 ± 0.025	0.280 ± 0.025	0.282 ± 0.025	0.464
MEDJL/FW	0.327 ± 0.024	0.325 ± 0.024	0.329 ± 0.024	0.209
MEPJL/FW	0.390 ± 0.022	0.389 ± 0.022	0.391 ± 0.023	0.431
ATDJL/FW	0.558 ± 0.020	0.557 ± 0.021	0.559 ± 0.019	0.710
ATPJL/FW	0.313 ± 0.028	0.310 ± 0.029	0.315 ± 0.026	0.203

*p* was compared between genders

*FW* femoral width, *AT* adductor tubercle, *ME* medial epicondyle, *LE* lateral epicondyle, *TT* tibial tubercle, *FH* fibular head, *IPP* the inferior pole of the patella, *DJL* distance from landmarks to the distal joint line, *PJL* distance from landmarks to posterior joint line

### Linear regression analysis

**Fig. 2:**
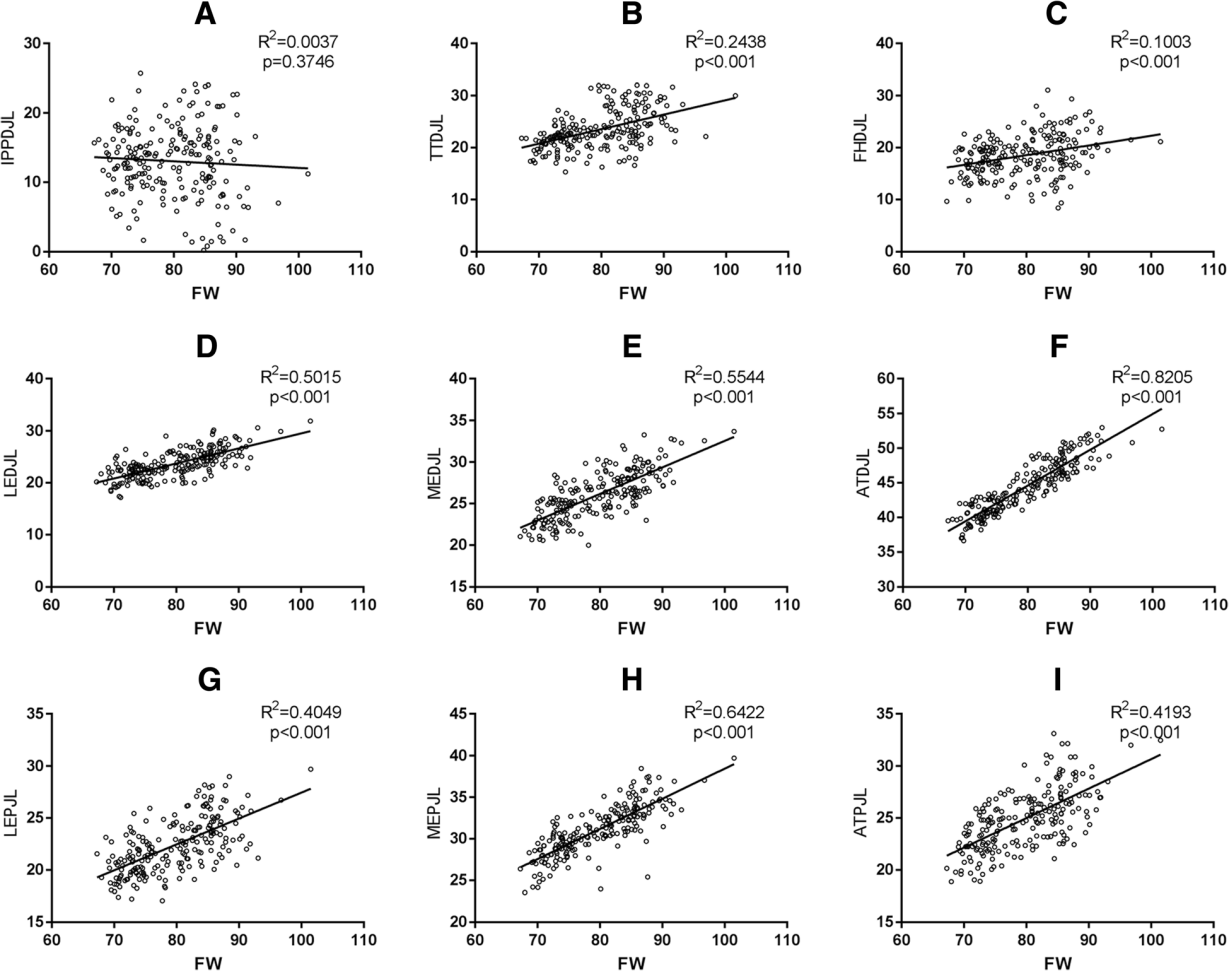
Correlation analysis on different landmarks. Correlation analysis between FW and distances from landmarks to distal or posterior joint line was performed (**a**-**i**). FW femoral width, AT adductor tubercle, ME medial epicondyle, LE lateral epicondyle, TT tibial tubercle, FH fibular head, IPP the inferior pole of the patella, DJL distal joint line, PJL posterior joint line

ATDJL showed the best linear co-relationship with FW (*R*^2^ = 0.8205), followed by MEDJL (*R*^2^ = 0.5544) and LEDJL (*R*^2^ = 0.5015). As for PJL degrees flexion of the knee, MEPJL showed the best linear co-relationship with FW (*R*^2^ = 0.6422), followed by ATPJL (*R*^2^ = 0.4193) and LEPJL (*R*^2^ = 0.4049). A not good co-relationship was showed for TTDJL (*R*^2^ = 0.2438) and FHDJL (*R*^2^ = 0.1003). No co-relationship between IPPDJL and FW had been found (*p* = 0.3746) (Fig. 2).

### Estimated distances

**Fig. 3:**
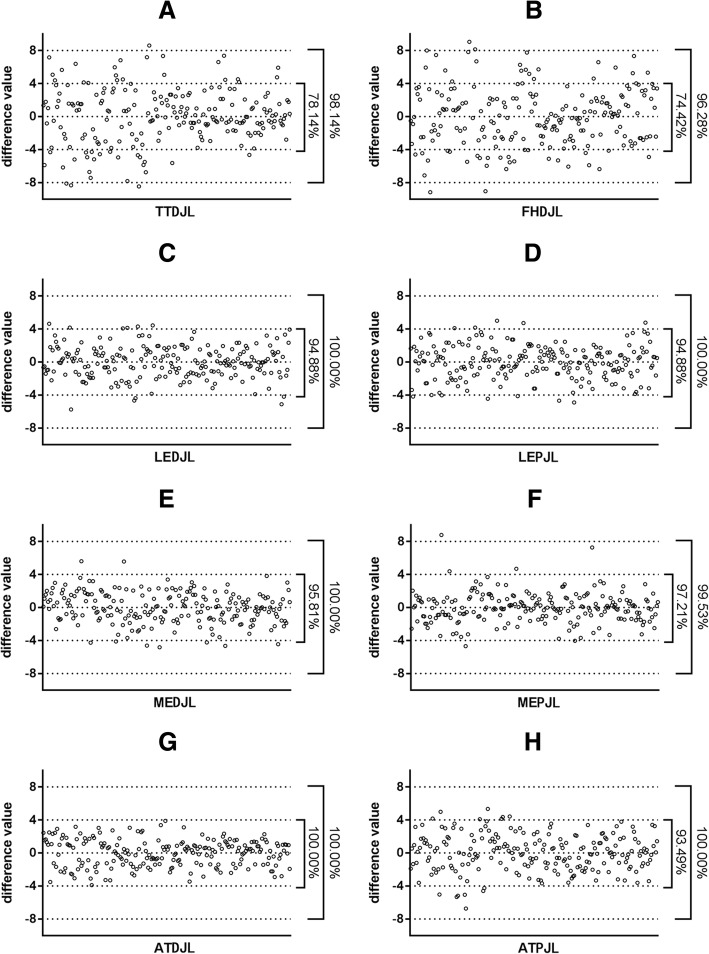
Difference between the measured distance and the estimated distance calculated by ratios. Difference values between the measured distance from landmarks to the joint line, and the estimated distance calculated from FW and mean ratios were calculated. Percentages of difference value within 4 mm or 8 mm were given on the graphs (**a**-**h**). FW femoral width, AT adductor tubercle, ME medial epicondyle, LE lateral epicondyle, TT tibial tubercle, FH fibular head, IPP the inferior pole of the patella, DJL distal joint line, PJL posterior joint line

All difference values between measured distances and estimated distances from reliable landmarks (LE, ME, AT) lain in a 8-mm threshold, except for one value from MEPJL (99.53%). Most of the difference values lain in a 4-mm threshold for MEDJL (95.81%), LEDJL (94.88%), MEPJL (97.21%), LEPJL (94.88%) and ATPJL (93.49%) except for ATDJL (100%) (Fig. 3).

### Difference between races

Ratios from different races were listed in Table 7. Unpaired *t* test was used to verify the differences between Chinese and other races and the results showed significant differences between the Chinese and other countries’ researches (*p* < 0.001). No statistical difference has been found amongst the Chinese population when compared with another research based on Chinese people.

**Table 7:** Ratios difference on races

	Our data (*n* = 215)	Servien et al. [10]	Ozkurt et al. [11]	Luyckx et al. [31]	Iacono et al. [28]	Xiao et al. [14]
TTDJL/FW	0.295 ± 0.040	0.27 ± 0.03				
LEDJL/FW	0.297 ± 0.024	0.28 ± 0.02	0.28 ± 0.02	0.32 ± 0.029		
LEPJL/FW	0.281 ± 0.025	0.29 ± 0.03	0.29 ± 0.03			
MEDJL/FW	0.327 ± 0.024	0.34 ± 0.02	0.35 ± 0.03	0.32 ± 0.027		
MEPJL/FW	0.390 ± 0.022	0.34 ± 0.03	0.34 ± 0.02			
ATDJL/FW	0.558 ± 0.020			0.52 ± 0.029	0.53 ± 0.03	*0.56 ± 0.03*

*p* was compared between ratios from different races. The significant difference had been found except for the ratio from the study of Xiao et al

*FW* femoral width, *AT* adductor tubercle, *ME* medial epicondyle, *LE* lateral epicondyle, *TT* tibial tubercle, *FH* fibular head, *IPP* the inferior pole of the patella, *DJL* distance from landmarks to the distal joint line, *PJL* distance from landmarks to posterior joint line

## Discussion

The current study gave answers as follows: (1) no side of the leg difference lain in these methods of determining JL positions amongst Chinese people, and gender differences were not found to be significant (except for IPP). (2) AT may be the best landmark to locate JL positions at full extension with the ratio of 0.558 in the Chinese population followed by ME (0.327) and LE (0.297). As for PJL degrees flexion, ME (0.390) may be the best choice for Chinese people followed by AT (0.313) and LE (0.281). (3) There are significant differences between the ratios of the Chinese population and other races.

Although restoring normal JL is necessary for either primary and revision TKAs, there is still no consensus on how to approach the normal JL [9]. During primary TKAs, surgeons could estimate the normal JL position based on the thickness of the femoral osteotomy. But when it comes to revision TKAs, normal anatomy had already been affected by primary TKA so surgeons cannot directly use the tangent line of the distal medial and lateral femoral condyles as JL. Leveling the position of the femoral prosthesis from primary TKA intraoperatively is inappropriate by reasons for loosening or the situation that JL position was already altered in primary TKA, which happened a lot [2]. Additionally, with the help of probably bone loss while removing the components from primary TKA or two stages of revision TKA after infection, reliable references to locate JL is needed during revision TKA. Using anatomical landmarks to locate JL position is well accepted during clinical practice [10]. Landmarks can be divided into two kinds, osteal landmarks and soft tissue landmarks. Soft tissue landmarks such as meniscal scar can be variable and not so distinct intraoperatively [17], whereas osteal landmarks are more reliable during surgery. Famously used osteal landmarks included adductor tubercle, medial and lateral epicondyles, tibial tubercle, fibular head and inferior patellar pole [10, 16, 18]. Surgeons can evaluate these landmarks in imageological examinations preoperatively and palpation intraoperatively.

With the help of those reliable landmarks, surgeons can measure the distance between the landmarks and JL from imageological examinations taken before the primary TKA, or from the contralateral knee if it is still intact with no TKA or fracture. But apparently, the usage of this method is limited when the images of previous examinations could not be found. Several credos like “two fingerbreadths of the tibial tubercle”, “20 mm above the fibular head” and “at the inferior patellar pole in extension” were used by some surgeons [19, 20], but with no literature to support those. And with the concept of slight changes in JL position could cause much worse outcomes in mind [3, 19, 20], these credos are too obscure to be used intraoperatively. So, we call for accurate methods that can apply to mostly (hopefully all) of the knee undergoing revision TKA. In order to achieve that goal, several studies measure the absolute distance from landmarks to JL [18, 21], but their results showed a variation in different ages, genders, body mass indices and races [10, 16, 18]. Servien et al. described a ratio between FW and the distances from LE or ME to JL and discovered a much smaller variation [10]. The usage of ratio had been proved to be more reliable [22] and negate those variations caused by age, body mass index and gender and showed reproducibility both from imageological examinations and intraoperative measurements [11, 23, 24]. However, differences still exist between different races, and previous studies had shown differences in anatomy between Chinese people and others [12–14]. Under this circumstance, we decided to provide methods to locate JL position in TKAs that suit the Chinese population.

The reason we chose CT scans for this study was that we could do the measurement based on a CAD model created by three-dimensional reconstruction, which should be the most similar way using imageological examination to stimulate measurement intraoperatively. Although researches showed no difference in radiographic MRI and CT measurements [25, 26], we preferred measuring the distance from the CAD model better. Based on our experiences with those measurements, landmarks may not lie in the same plane that parallels to the coronal plane according to JL. Under this circumstance, measurements took place on MRI may not be equal to the real distances. No statistical difference had been found amongst the measurements of intraobserver and interobserver (Tables 1 and 2), demonstrating the methods’ reproducibility. As for ratios calculation, some researchers may prefer femoral diameter rather than FW [24]. However, research based on Chinese population showed that FW had a better linear correlation with ATDJL than femoral diameter [14], so we decided to use FW to calculate the ratios.

No difference was found between distances between the left and right knee amongst bilateral CT scan and total samples. Our results came in line with Havet et al. [18]. By the help of this statistical evidence, the measurement could be used in revision TKAs while the contralateral knee is free from TKA, fracture or osteoarthritis.

Gender differences were found to be significant on absolute distances except for IPPDJL, taking together with statistical gender difference in the ratio of IPPDJL, which may probably be explained by the variability of patella position [3, 4, 27]. In this case, we believed that IPP may not be a good reference to locate JL in the Chinese population. Most of the other researches showed no gender discrepancy in ratios of landmarks and JL [10, 11]. We came in line with those researchers. There is still a need for further research to rectify our results.

AT is the attachment point of the adductor muscle and is easy to be found during revision surgeries. Iacono et al. firstly used AT as landmarks to determine the JL and demonstrate its repeatability and accuracy preoperatively and intro-operatively [16, 28]. Recently, Xiao et al. proved that AT is a reliable landmark in the Chinese population [14]. In our study, we drew the same conclusion. ATDJL/FW showed the highest *R*^2^ (0.8205) above all those landmarks, which indicates AT may be the most precise landmarks in locating JL on full extension knee in Chinese people. Putting these all together, AT may be the first choice for surgeons to determine the level of the JL.

ME and LE had been used for years as landmarks in researches [2, 19]. Researchers used the most prominent point of the LE to measure the distance but as for ME, there are two methods. One is the most prominent point of medial epicondyle while the other is the sulcus of the medial epicondyle. The sulcus of medial epicondyle may be less accurate in an arthritic deformed knee [29], so we chose to use the most prominent point. In another way, we believed that the most prominent point may be easier to locate and more accurate during palpation intro-operatively. Our data showed ME and LE are less precise than AT based on relatively lower *R*^2^, but still they had a relatively strong correlation amongst distances to distal JL and FW in the Chinese population. These results are consistent with previous researches [9, 16]. Hence, ME and LE may serve as second choices while AT is not available.

TT is the attachment point of the patellar tendon and is palpable during surgery. Many investigators have studied this landmark and came out with different conclusions. Servien et al. [10] believed that TT is a precise landmark while as Bieger et al. [22] and Mason et al. [19] hold discordant thoughts. In normal Chinese population, our data revealed *R*^2^ = 0.2438, which means that TT may not be a preferred landmark to level the distal normal JL. Nevertheless, TT is more like a small area rather than a precise point due to the cover of the patellar tendon, which undoubtedly limited its usage.

FH may not be a precise landmark in locating femoral JL, which had been proved by several investigators recently [15, 18]. In Chinese people, FH may not serve as a reliable landmark either (*R*^2^ = 0.1003). Otherwise, the anatomical position of the FH is always various [10] and untouchable during revision surgeries unless surgeons would like to take the risk of damaging the surrounding structure, not alone primary TKA.

During revision TKAs, surgeons always need to locate the tibial JL at PJL degrees first. In our study, with the difficulty to get CT data while knee joint is in PJL degrees flexion, we decided to use posterior femoral JL to represent tibial JL just like other researchers did [10, 11]. Regression analyses based on our data showed that AT, ME and LE have good pertinence during the leveling of the PJL degrees flexion JL. No study reported the usage to locate the posterior JL by the help of AT, but investigators had already proved that ME and LE can be used to level the posterior JL amongst other races [10, 11]. We held the same thoughts with these investigators.

JL positions altered within 4–8 mm may lead to postoperative complications like pain or lack of range of movement [5, 8, 30]. In order to further verify the accuracy of those landmarks, we used the ratios and FW to calculate the distance between JL and those landmarks. And if the deviations from one landmark are within 8 mm, 4 mm even better, then this landmark should be considered accurate. After data processing, using ratios of AT to calculate JL position proved to be the most accurate method of Chinese people. Other landmarks (ME and LE) could satisfy the surgical need in most situations.

When compared with the ratios of other races’ researches, significant differences had been found amongst our data based on the Chinese population and other races’ data [10, 11, 14, 28, 31]. Otherwise, we found no statistical difference that exists on our data onto ATDJL and ratios from Xiao et al. based on the Chinese population. In a way, our data may prove that it is necessary to come up with a set of ratios for different races.

## Conclusions

Our study has demonstrated that AT, ME and LE can be used as veracity and reliable landmarks to locate the normal JL. Differences should be noticed between different races, and it may serve better effect using ratios based on the Chinese population when TKA was operated on Chinese people.

## Abbreviations

AT: Adductor tubercle
CT: Computed tomography
FH: Fibular head
FW: Femoral width
IPP: The inferior pole of the patella
JL: Joint line
LE: Lateral epicondyle
ME: Medial epicondyle
TKA: Total knee arthroplasty
TT: Tibial tubercle
